# Effect of nutrition impact symptoms on oral nutritional supplements energy intake and use days in patients with head and neck cancer: A cross‐sectional study

**DOI:** 10.1002/cam4.7288

**Published:** 2024-05-21

**Authors:** Tingting Dai, Jinli Xian, Xuemei Li, Zhiqiang Wang, Wen Hu

**Affiliations:** ^1^ Department of Clinical Nutrition, West China Hospital Sichuan University Chengdu Sichuan China; ^2^ Department of Clinical Nutrition, MianYang Central Hospital Mianyang Sichuan China

**Keywords:** head and neck cancer, nutrition impact symptoms, ONS energy intake, ONS use days, Oral nutritional supplements

## Abstract

**Background:**

This study aims to explore the effect of nutritional impact symptoms (NIS) on oral nutritional supplements (ONS) energy intake and use days among head and neck cancer (HNC) patients.

**Methods:**

A cross‐sectional study was conducted among HNC patients in a hospital in western China between January 2019 and June 2020. The NIS was from the Patient‐Generated Subjective Global Assessment (PG‐SGA) scale. Mann–Whitney test was used to examine the differences between different kinds of NIS and ONS use days. Binary logistic regression was used to determine the effect of NIS on ONS energy intake.

**Results:**

The most prevalent four NIS were no appetite (35.3%), dysphagia (29.4%), vomiting (13.2%) and oral pain (12.5%), respectively. All patients in the study were malnutrition. Patients with xerostomia or oral pain had less ONS use days than those without these symptoms. Patients with vomiting (OR 0.09, 95% CI 0.02–0.50) or pain (OR 0.15, 95% CI 0.02–0.89) were less likely to have ONS energy intake ≥400 kcal/day than those without these symptoms after adjusting the confounding factors. In addition, one‐point increase in total NIS score was associated with a lower proportion of ONS energy intake ≥400 kcal/day (OR 0.77, 95% CI 0.59–0.99).

**Conclusion:**

Xerostomia, oral pain, vomiting and pain should be strengthened and intervened to improve ONS use and nutritional status among HNC patients with malnutrition.

## 1 INTRODUCTION

Among head and neck cancer (HNC) patients, approximately 35%–60% are malnourished at diagnosis because of tumor burden, reduced dietary intake, or cancer‐related anorexia and cachexia.^1^ The incidence of malnutrition increases throughout treatment.^2^ Malnutrition is associated with increased morbidity and mortality in HNC patients.^3^

Nutritional impact symptoms (NIS) are adverse symptoms that affect patients' oral intake.^4^ NIS burden is a risk factor for reduced dietary intake, weight loss, and survival.^5^ HNC patients could obtain a significant burden of NIS before chemoradiotherapy,^5^ and the NIS score of HNC patients would increase during the treatment.^6^ NIS due to head and neck chemoradiotherapy is common in HNC patients, which includes dysphagia, xerostomia, trismus, salivary issues, mucositis, and oral pain.^7^ These NIS may cause pain and inflammatory responses, thereby limiting energy intake, increasing stress responses, leading to weight loss and malnutrition, and even psychological problems.^8^, ^9^ Studies have shown that the severity degree of NIS during treatment was positively associated with weight loss in HNC patients.^10^

In ESPEN guideline, oral nutritional supplementations (ONS) was defined as “Supplementary oral intake of dietary food for special medical purposes in addition to the normal food.”^11^ ONS is a nutritional liquid, semi‐solid or powder that provides a variety of macronutrients and micronutrients for the purpose of increasing oral nutrient intake.^12^ It is needed for people whose dietary energy intake is not expected to reach more than 60% of the recommended energy intake over 10 days, and should be at least 400 to 600 kcal/day in addition to dietary intake.^13^ ONS has a lot of benefits for people with nutritional risk, such as increasing the body mass, reducing the incidence of mortality and complications, and reducing the proportion of hospital readmission.^12^ It can also improve dietary energy intake, nutritional status and offer better tolerance to treatment of cancer patients.^6^, ^14^, ^15^, ^16^, ^17^ It is the preferred nutritional treatment for patients with malnutrition or nutritional risk without enteral nutritional contraindications.^12^, ^18^ In addition, ONS use among HNC patients, particularly during chemo‐and/or radiotherapy, is recommended by ESPEN.^19^

NIS is an important factor influencing the requirement for nutrition support,^20^ and patients with higher NIS score may have higher requirement for nutrition support.^15^ A previous study found that the NIS score during radiotherapy was positively associated with ONS energy and protein intake in HNC patients.^6^ Most patients with NIS burden tend to choose ONS as meal replacements.^6^, ^15^ Most ONS is liquid or powder, which may produce less satiety than solid foods and can be easier to ingest.^21^

Understanding dietary issues and NIS management, as well as the relationship with appropriate nutritional support strategies, could help to develop intensive nutrition management to improve the nutritional status in HNC patients. A previous study has analyzed the correlation between total NIS score and ONS energy and protein intake in Malaysia.^6^ However, there was a rare study of NIS and ONS intake in China. Therefore, this study aims to explore the effect of different kinds of NIS on nutrition support in China.

## 2 MATERIALS AND METHODS

### 2.1 Study design

This study is a cross‐sectional study. This study included inpatients diagnosed with HNC at West China Hospital, Sichuan University in western China between January 2019 and June 2020.

### 2.2 Sample size calculation

A previous study found that the total NIS interference scores had significant difference between Low ONS intake and high ONS intake group (33 ± 13 vs. 44 ± 11; *p* < 0.01).^15^ According to the formula of sample size calculation:

$$n=\frac{2\sigma^{2}{\left(Z_{\alpha /2}+Z_{\beta }\right)}^{2}}{{\left(u_{1}-u_{2}\right)}^{2}}$$

We set $Z_{\alpha /2}=1.96$, $Z_{\beta }=1.28$, σ=11, $u_{1}=44$, $u_{2}=33$, the calculated sample size was 42. In the study, the actual total sample size included 136 patients.

### 2.3 Participants

Eligibility criteria for patients were as follows: (a) nutritional risk screening (NRS) 2002 scores ≥3; (b) can take food orally; (c) agree to use ONS; (d) before treatment of chemotherapy/radiotherapy/chemoradiotherapy, or before a new cycle treatment of chemotherapy/radiotherapy/chemoradiotherapy; (d) have no history of mental or psychological disease, hematological diseases, and other malignant tumors. The Nutritional Risk Screening tool (NRS‐2002) was generally conducted within 24 h of admission to screen patients for nutritional risk.^22^ Patients aged ≤18 years (*n* = 1) and had missing information of height (*n* = 1) were excluded in the study. A total of 136 subjects were included in the analysis. In addition, written informed consent for processing personal data was obtained from each participant.

### 2.4 Variables

#### 2.4.1 Exposure variable: NIS

The Patient‐Generated Subjective Global Assessment (PG‐SGA) scale was generally carried out within 48 h of admission to assess the patient for malnutrition.^22^ The PG‐SGA scale is a subjective overall nutritional status rating scale that is specialized for cancer patients.^23^, ^24^ The PG‐SGA scale was assessed by nutritionists in the Department of Clinical Nutrition in the hospital. A higher PG‐SGA score indicated poorer nutritional status.^25^ Patients were categorized as well‐nourished, moderately or suspected of being malnourished or severely malnourished upon completion of the assessment.^26^ The NIS was from the PG‐SGA scale, including “no dietary problem” (scored 0 point), “no appetite, don't want to eat” (scored 3 point), “nausea”(scored 1 point), “vomit”(scored 3 point), “constipation”(scored 1 point), “diarrhea”(scored 3 point), “oral pain”(scored 2 point), “xerostomia”(scored 1 point), “no taste or abnormal taste”(scored 1 point), “food odor interference”(scored 1 point), “dysphagia”(scored 2 point), “early satiety”(scored 1 point), “pain”(scored 3 point), and “other (e.g., emotional low, money or tooth problems)”(scored 1 point).^26^ Each of NIS will input into “Yes” or “No.” The total score of NIS was added by the above 14 NIS, and the total NIS score was recoded into tertiles (low, medium, and high) in the study.

#### 2.4.2 Outcome variable: ONS energy intake and ONS use days

The patients used ONS when they were assessed as having nutritional risk (NRS‐2002 score ≥3 points) or malnutrition (PG‐SGA score ≥4 points) without enteral nutritional contraindications.^12^, ^18^, ^26^ And the ONS use obtained the consent of the patients and (or) their family. The nutritionists assessed the patients' nutrition requirements, dietary intake, gastrointestinal digestion and absorption capacity through dietary investigation and assessment, physical measurement, clinical examination and laboratory examination.^27^ Then, the personalized ONS programs for different patients were made by the nutritionists. Early ONS support was carried out carefully, which usually started with small doses and gradually increased according to the patient's tolerance.^12^ The ONS should be used until the patient was able to resume normal eating or refused to continue using ONS.^12^ The ONS energy intake of each patient was computer automatically recorded after the ONS prescription was entered by the nutritionist in the nutrition management system on computer every day. The ONS was given in the form of individually packaged powder or liquid, and it is usually taken between meals. According to an expert consensus in China, it is recommended that ONS should be at least 400–600 kcal/day in addition to dietary intake.^13^ Thus, the average ONS energy intake was categorized as ≥400 and <400 kcal/day in this study.

#### 2.4.3 Covariates

The patients' age, sex, tumor‐related surgery history, tumor recurrence or metastasis, smoking history, drinking history, neoplasm staging, tumor site, length of hospital stay and whether leaving the hospital with ONS were collected from the medical records by the researchers. Body mass index (BMI) was calculated as weight (kg)/height^2^ (m^2^), and divided into four categories according to the Chinese standard.^28^ Physical activity in the last month was assessed using the Eastern Cooperative Oncology Group (ECOG) performance status assessment standard, which contained five grades. Grade 1 refers “normal with no limitations”; grade 2 refers “not my normal self, but able to be up and about with fairly normal activities”; grade 3 refers “not feeling up to most things, but in bed or chair less than half the day”; grade 4 refers “able to do little activity and spend most of the day in bed or chair”; and grade 5 refers “pretty much bed ridden, rarely out of bed.”^24^ Comorbidities were quantified by using the age‐adjusted Charlson comorbidity index (ACCI).^29^ In the study, tumor site included nasopharynx, hypopharynx, larynx, tongue, tonsil, primary unknown cervical lymph node metastatic squamous cell carcinoma, soft tissue sarcoma, gingival and others.

### 2.5 Data analysis

Descriptive statistics were used for the sample characteristics. The categorical variables were described using frequency and percentage, and the continuous variables were described using mean and standard deviation (SD). Mann–Whitney test was used to examine the differences between different kinds of NIS and ONS use days. Kruskal–Wallis test was used to examine the differences between the degree of NIS and ONS use days. To determine the effect of NIS on ONS energy intake (<400 and ≥400 kcal/day), odds ratios (ORs) and 95% confidence intervals (CIs) for the outcome variables were calculated using binary logistic regression. Two models were used: model 1 was unadjusted, and model 2 was adjusted for age, sex, BMI, weight change in the last 2 weeks, physical activity in the last month, treatment regimen, tumor‐related surgery history, tumor recurrence or metastasis, ACCI, smoking history, drinking history, neoplasm staging, length of hospital stay and tumor site. The Pearson goodness‐of‐fit test indicated that all logistic regression models used in this study were good fits (*p* > 0.05).

All analyses were performed using STATA software (Version 15, StataCorp, College Station, TX, USA). Statistical significance was considered when *p* < 0.05 (two‐sided).

## 3 RESULTS

Table 1 presented the basic characteristics of the participants. Among the HNC patients in the study, the mean age was 48.6 (SD 14.3) years old, and 60.3% of them were male. The mean ACCI of the patients was 7.1. There were 64.0% of the patients with the nasopharynx cancer, 11.8% with hypopharyngeal cancer, 5.9% with laryngocarcinoma. Among the patients, 16.9% had a tumor‐related surgery, and 82.4% were primary tumor with metastasis. There were more than half of the patients in stage IV of tumor, and 68.4% having a decreased weight in the last 2 weeks. All patients in the study were malnutrition, with 74.3% assessed as severely malnourished. The proportion of patients obtaining ONS energy intake ≥400 kcal/day was 76.5% and the mean of ONS use days was 4.7 (SD 4.2). In addition, the mean of length of hospital stay was 10.5 (SD 6.8). There were 25.0% of the patients leaving the hospital with ONS.

**TABLE 1:** Basic characteristics of patients with head and neck cancer (*N* = 136).

Independent variables	Total population
Age (mean ± SD), years old	48.6 ± 14.3
Gender (*n*, %)	
Male	82 (60.3)
Female	54 (39.7)
BMI (*n*, %)	
Underweight	41 (30.1)
Normal	70 (51.5)
Overweight	23 (16.9)
Obese	2 (1.5)
Weight change in the last 2 weeks^a^ (*n*, %)	
Decrease	91 (68.4)
Unchange	34 (25.6)
Increase	8 (6.0)
Overall physical activity in last month (*n*, %)	
Grade 1	36 (26.5)
Grade 2	80 (58.8)
Grade 3	14 (10.3)
Grade 4	5 (3.7)
Grade 5	1 (0.7)
Nutritional status (*n*, %)	
Moderate malnutrition	35 (25.7)
Severe malnutrition	101 (74.3)
ONS use days (mean ± SD), days	4.7 ± 4.2
ONS energy intake (kcal/day) (*n*, %)	
<400	32 (23.5)
≥400	104 (76.5)
Treatment regimens (*n*, %)	
Radiotherapy	14 (10.3)
Chemotherapy	97 (71.3)
Chemoradiotherapy	25 (18.4)
Tumor‐related surgery history (*n*, %)	
No	113 (83.1)
Yes	23 (16.9)
Tumor recurrence or metastasis (*n*, %)	
Primary tumor without metastasis	20 (14.7)
Recurrent	4 (2.9)
Primary tumor with metastasis	112 (82.4)
ACCI (mean ± SD)	7.1 ± 2.0
Smoking history (*n*, %)	
Yes	41 (30.1)
No	95 (69.9)
Drinking history (*n*, %)	
Yes	37 (27.2)
No	99 (72.8)
Neoplasm staging (*n*, %)	
II	7 (5.1)
III	28 (20.6)
IV	71 (52.2)
Unstaged	30 (22.1)
Length of hospital stay (mean ± SD)	10.5 ± 6.8
Tumor site (*n*, %)	
Nasopharynx	87 (64.0)
Hypopharynx	16 (11.8)
Larynx	8 (5.9)
Tongue	5 (3.7)
Tonsil	4 (2.9)
Primary unknown cervical lymph node metastatic squamous cell carcinoma	4 (2.9)
Soft tissue sarcoma	4 (2.9)
Gingival	3 (2.2)
Others	5 (3.7)
Leaving the hospital with ONS (*n*, %)	
Yes	34 (25.0)
No	102 (75.0)

^a^ Missing value 3.

Table 2 showed the differences between NIS and ONS use days among the patients. The four prevalent NIS were no appetite (35.3%), dysphagia (29.4%), vomiting (13.2%) and oral pain (12.5%), respectively. The low, medium and high degree of the total scores of NIS were 39.7%, 35.3%, 25.0%, respectively. Patients with xerostomia had less ONS use days than those without the symptom (*p* < 0.05). And patients with oral pain had less ONS use days than those without the symptom (*p* < 0.01).

**TABLE 2:** Differences between NIS and ONS use days among the patients with head and neck cancer (*n* = 136).

Independent variables	*N* (%)	ONS use days (mean ± SD)	*p*‐value
No dietary problem			
No	122 (89.7)	4.6 ± 4.3	0.13
Yes	14 (10.3)	5.6 ± 3.7	
No appetite, don't want to eat			
No	88 (64.7)	4.8 ± 4.5	0.95
Yes	48 (35.3)	4.5 ± 3.7	
Nausea			
No	121 (89.0)	4.9 ± 4.4	0.32
Yes	15 (11.0)	3.3 ± 2.2	
Vomit			
No	118 (86.8)	4.8 ± 4.4	0.64
Yes	18 (13.2)	3.9 ± 2.9	
Constipation			
No	128 (94.1)	4.7 ± 4.2	0.78
Yes	8 (5.9)	4.9 ± 4.9	
Diarrhea			
No	135 (99.3)	4.7 ± 4.3	0.83
Yes	1 (0.7)	3.0 ± 0	
Oral pain			
No	119 (87.5)	5.0 ± 4.3	<0.01
Yes	17 (12.5)	2.6 ± 2.8	
Xerostomia			
No	133 (97.8)	4.8 ± 4.3	<0.05
Yes	3 (2.2)	1.0 ± 0.0	
No taste or abnormal taste			
No	121 (89.0)	4.6 ± 4.2	0.92
Yes	15 (11.0)	5.1 ± 4.5	
Food odor interference			
No	127 (93.4)	4.8 ± 4.3	0.89
Yes	9 (6.6)	3.8 ± 2.1	
Dysphagia			
No	96 (70.6)	4.4 ± 4.3	0.08
Yes	40 (29.4)	5.3 ± 4.1	
Early satiety			
No	122 (89.7)	4.7 ± 4.0	0.70
Yes	14 (10.3)	4.9 ± 5.9	
Pain			
No	123 (90.4)	4.8 ± 4.4	0.45
Yes	13 (9.6)	3.6 ± 2.7	
Other (e.g., emotional low, money or tooth problems)			
No	121 (89.0)	4.7 ± 4.1	0.77
Yes	15 (11.0)	4.7 ± 5.4	
Degree of NIS			
Low	54 (39.7)	5.5 ± 5.1	0.22
Medium	48 (35.3)	3.9 ± 3.4	
High	34 (25.0)	4.5 ± 3.7	

In Table 3, when adjusting for the confounding factors, patients with vomiting (OR 0.09, 95% CI 0.02–0.50) or pain (OR 0.15, 95% CI 0.02–0.89) were less likely to have ONS energy intake ≥400 kcal/day than those without these symptoms, respectively. In addition, one‐point increase in total NIS score was associated with a lower proportion of ONS energy intake ≥400 kcal/day (OR 0.77, 95% CI 0.59–0.99). However, other NIS was not associated with ONS energy intake in the study.

**TABLE 3:** Logistic regression analyses of the association between the NIS and ONS energy intake (<400 kcal/day vs. ≥400 kcal/day) among the patients with head and neck cancer.

Independent variables	Model 1^a^	Model 2^b^
	OR (95% CI)	OR (95% CI)
No dietary problem		
No	Reference	Reference
Yes	4.43 (0.56–35.25)	2.81 (0.21–37.26)
No appetite, don't want to eat		
No	Reference	Reference
Yes	0.62 (0.28–1.40)	1.54 (0.43–5.51)
Nausea		
No	Reference	Reference
Yes	0.30 (0.10–0.90)*	0.39 (0.09–1.59)
Vomit		
No	Reference	Reference
Yes	0.32 (0.11–0.90)*	0.09 (0.02–0.50)**
Constipation		
No	Reference	Reference
Yes	0.92 (0.18–4.79)	1.47 (0.11–19.26)
Oral pain		
No	Reference	Reference
Yes	0.38 (0.13–1.10)	0.30 (0.04–2.10)
Xerostomia		
No	Reference	Reference
Yes	0.15 (0.01–1.66)	0.42 (0.03–6.68)
No taste or abnormal taste		
No	Reference	Reference
Yes	2.14 (0.46–10.04)	7.85 (0.86–71.95)
Food odor interference		
No	Reference	Reference
Yes	0.35 (0.09–1.41)	0.20 (0.02–1.66)
Dysphagia		
No	Reference	Reference
Yes	1.09 (0.45–2.61)	1.01 (0.31–3.30)
Early satiety		
No	Reference	Reference
Yes	0.74 (0.22–2.56)	0.84 (0.16–4.36)
Pain		
No	Reference	Reference
Yes	0.45 (0.14–1.49)	0.15 (0.02–0.89)*
Other (e.g., emotional low, money or tooth problems)		
No	Reference	Reference
Yes	2.14 (0.46–10.04)	2.96 (0.47–18.83)
Degree of NIS		
Low	Reference	Reference
Medium	0.40 (0.14–1.11)	0.38 (0.09–1.60)
High	0.27 (0.09–0.79)*	0.23 (0.05–1.05)
Total NIS scores	0.77 (0.64–0.93)**	0.77 (0.59–0.99)*

* *p* < 0.05;

** *p* < 0.01.

^a^ Model 1: unadjusted.

^b^ Model 2: adjusted for age, sex, BMI, weight change in the last 2 weeks, physical activity in the last month, treatment regimens, tumor‐related surgery history, tumor recurrence or metastasis, ACCI, smoking history, drinking history, neoplasm staging, length of hospital stay and tumor site.

## 4 DISCUSSION

In this study, the incidence of NIS was 89.7%, and the highest proportion of NIS was no appetite (35.3%), followed by dysphagia (29.4%), vomiting (13.2%) and oral pain (12.5%), which is similar to previous studies.^5^, ^30^, ^31^ These symptoms can lead to malnutrition and weight loss, with a potential risk of malabsorption.^32^ Studies have shown that loss of appetite had a great influence on dietary intake in HNC patients.^5^, ^10^ And radiation‐related symptoms such as dysphagia and oral mucositis were positively associated with the rate of weight loss.^10^ A study in Sichuan province in China found that cancer patients tend to report that nausea makes them more miserable, but vomiting is more likely to require intervention.^33^

There were about 20% of the HNC patients had a tumor‐related surgery, and 82.4% of the patients were primary tumor with metastasis. In addition, more than half of the patients were in stage IV of tumor. Notably, malnutrition was present in all patients, with 74.3% assessed as severely malnourished. However, patients with ONS energy intake ≥400 kcal/day only accounted for 76.5%. This result may be due to low compliance, taste fatigue, and high cost of ONS use in cancer patients.^34^, ^35^ Additionally, there was a high prevalence of having a decreased weight in the last 2 weeks in the patients. In a previous study, patients with critical weight loss during radiotherapy had worse disease‐specific survival related to poor response to treatment.^6^ Weight maintenance can lead to beneficial outcomes and is an appropriate aim for nutritional interventions. Therefore, weight monitoring is very important during treatment. Improving the use of ONS will be important to help the weight maintenance and improve the nutritional status of HNC patients.

HNC patients with xerostomia had less ONS use days than those without the symptom in this study. Previous studies have found that xerostomia was severe enough to cause eating or drinking problems.^36^, ^37^ And a previous study found that xerostomia was associated with reduced dietary intake.^5^ Patients with xerostomia would change their diet and prefer foods that could be eaten easily.^38^ They would have less desire to eat starch and dry foods such as bread and pastries.^38^ However, patients with xerostomia may consider that xerostomia is not severe enough to require ONS, as they could try to change their diet such as choosing moist foods, increasing fluid consumption and so on.^39^ In addition, patients with oral pain had less ONS use days than those without this symptom. Oral mucositis refers to erythematous and/or ulcerative lesions of the oral mucosa^40^ and is a frequent and severe consequence of chemotherapy or radiotherapy to the head and neck.^41^ Pain is a common side effect of mucositis, with the ulcerative stage being especially painful.^42^ Ulceration of the oral mucosa and the resulting pain can impair a patient's ability to swallow and eat.^43^, ^44^ A study in 2011 among 60 HNC patients, oral ulceration was essentially absent after 1 month posttreatment completion.^45^ As mucosal injury continued to resolve, the oral intake and diet will be improved.^45^ Therefore, the method to repair and protect ulcerated oral mucosa without promoting cancer cell growth is important. Simultaneously, developing a kind of ONS with less irritating and more palatable for oral ulcer may be a good way to improve patients' nutrition intake. In addition, HNC patients usually experience various NIS at the same time,^36^, ^46^ and further study needs to explore the joint action of NIS and ONS use.

Patients with vomiting was less likely to have ONS energy intake ≥400 kcal/day than patients without this symptom. Vomiting belongs to the gastrointestinal symptoms, and was found significantly correlated with the weight loss rate.^10^ And this symptom is closely related to chemotherapy. The drugs can arouse vomiting by stimulating the neural pathway related to gastrointestinal function through neurotransmitters (e.g., serotonin and substance P) or directly influencing the nerve center.^47^ And vomiting is interlinked with patients' appetite and the feeling of fullness.^48^ In a recent cross‐sectional study among 1171 cancer patients in China, most patients believed that vomiting and pain need to be intervened.^33^ Therefore, it is recommended to continuously monitor the NIS of HNC patients when they have insufficient dietary intake during treatment.^49^ In the study, patients with pain were less likely to have ONS energy intake ≥400 kcal/day than those without the symptom. Pain affects more than 70% of HNC survivors.^50^ Chronic pain resulted from cancer or cancer treatments such as surgery, radiation, and chemotherapies or in combination, is particularly challenging as patients suffer from multiple oral complications such as orofacial pain, changes in taste, and oral dysfunction.^51^, ^52^ A pilot study in Brazil found that HNC patients experience a wide range of pain symptoms with altered mechanical, chemical, and temperature sensation.^50^ This may be a reason for decreased dietary and ONS intake of HNC patients with pain.

The study also found that one‐point increase in total NIS score was associated with a lower proportion of ONS energy intake ≥400 kcal/day. This is different from a previous study, which found a significant positive relationship between the total NIS score and ONS energy and protein intake at the middle of radiotherapy.^6^ A higher NIS burden may associate with more severe eating problems, a higher risk of malabsorption and less oral intake,^32^ which may reduce dietary and ONS intake of patients. Therefore, more studies about NIS and ONS intake are needed.

To the best of our knowledge, this is the first study to investigate the effect of different kinds of NIS on ONS energy intake and ONS use days in HNC patients. However, this study also has some limitations. First, this is a cross‐sectional study, which can't draw a causal inference. Second, patients' financial condition^53^ may influence the ONS intake, which should be included as a confounding factor in further study. Third, NIS was from the PG‐SGA scale and didn't include other head and neck symptoms, such as lack of energy, depression, difficulty chewing, thick saliva, anxiety and mucositis,^6^ which need to be considered in further study. Fourth, this study didn't include the objective blood test data regarding nutrition status. Previous studies have reported the effect of chemotherapy/radiotherapy/chemoradiotherapy on myelosuppression,^54^, ^55^, ^56^ anemia^57^ and serum albumin.^58^, ^59^ In another study, the PG‐SGA score ≥9 was the independent predictor of radiation esophagitis.^60^ As the blood test can be greatly affected by antitumor therapy,^54^, ^55^, ^56^, ^57^, ^58^, ^59^ the PG‐SGA scale may be more suitable to reflect the nutritional status of the HNC patients on admission. However, the including of blood test would reflect the nutritional status of patients more comprehensively and significantly add to the study. We will consider to include the blood test in further study to analyze the association between nutrition impact symptoms and nutritional supplements intervention in HNC patients with different nutritional status.

## 5 CONCLUSION

NIS is an important and convenient early warning indicator for malnutrition. HNC patients with xerostomia or oral pain had less ONS use days than those without these symptoms. Patients with vomiting or pain were less likely to have ONS energy intake ≥400 kcal/day than those without these symptoms. In addition, one‐point increase in total NIS score was associated with a lower proportion of ONS energy intake ≥400 kcal/day. Therefore, active measures should be taken to alleviate the NIS, particularly vomiting and pain, to improve the nutrition intake and nutritional status of HNC patients.

## AUTHOR CONTRIBUTIONS

**Tingting Dai:** Conceptualization (lead); data curation (lead); formal analysis (supporting); methodology (equal); project administration (lead); supervision (lead); writing – original draft (supporting); writing – review and editing (supporting). **Jinli Xian:** Conceptualization (equal); data curation (equal); formal analysis (lead); methodology (equal); supervision (equal); validation (lead); visualization (lead); writing – original draft (lead); writing – review and editing (lead). **Xuemei Li:** Conceptualization (supporting); data curation (supporting); formal analysis (supporting); methodology (supporting); supervision (supporting); writing – review and editing (supporting). **Zhiqiang Wang:** Data curation (supporting); supervision (supporting); writing – review and editing (supporting). **Wen Hu:** Conceptualization (equal); data curation (equal); formal analysis (supporting); methodology (equal); project administration (equal); supervision (equal); writing – review and editing (equal).

## FUNDING INFORMATION

This research was funded by Food Science and Technology Foundation of China Society of Food Science and Technology, Grant/Award Number: CAJJ‐2‐1.

## CONFLICT OF INTEREST STATEMENT

The authors declare that the research was conducted in the absence of any commercial or financial relationships that could be construed as a potential conflict of interest.

## ETHICS STATEMENT

The study protocol was approved by the ethics committee of West China Hospital, Sichuan University (No. 2019‐725).

## Data Availability

The data sets used and/or analyzed during the current study will be available from the corresponding author on reasonable request.
